# Regulatory mechanism of M2d tumor-associated macrophage-derived exosomal apolipoprotein E on platinum resistance in ovarian cancer

**DOI:** 10.3389/fonc.2026.1946625

**Published:** 2026-10-15

**Authors:** Chunmei Wang, Ranjun Wu, Genhai Zhu

**Affiliations:** Department of Gynecology, Hainan General Hospital, Affiliated Hainan Hospital of Hainan Medical University, Haikou, Hainan, China

**Keywords:** apolipoprotein E, ovarian cancer, platinum resistance, TAMs, xenograft models

## Abstract

**Background:**

Platinum-based chemotherapy plus cytoreductive surgery represents the standard first-line therapy for ovarian cancer (OC), which yields robust early therapeutic responses in most patients. However, platinum resistance is a formidable clinical hurdle, and how macrophages mediate drug resistance within the OC tumor microenvironment remains poorly understood.

**Methods:**

We cultured SKOV3 OC cells and THP-1 monocytes, induced THP-1 differentiation into M2d tumor-associated macrophages. Stable apolipoprotein E (ApoE) -overexpressing SKOV3 cell lines were constructed via lentiviral transfection and puromycin screening. We established nude mouse subcutaneous xenograft models with mixed tumor cells and M2d macrophages, administered cisplatin treatment and collected tumor tissues after sacrifice by cervical dislocation. RNA was extracted from tumor samples to conduct RT-qPCR for gene expression detection. Tumor tissue sections were prepared for HE staining, TUNEL apoptosis staining, and Western blot analysis to detect ApoE and phosphatidylinositol 3-kinase (PI3K)/AKT/mammalian target of rapamycin (mTOR) pathway protein expression.

**Results:**

Using nude mouse subcutaneous xenograft models, we demonstrated that M2d-type tumor-associated macrophages (M2d TAMs) promote platinum resistance and elevate tumoral ApoE levels. In OC xenografts with stable ApoE overexpression, cisplatin-induced apoptosis was profoundly repressed, resulting in reduced cisplatin sensitivity, while this inhibitory effect was not observed in the empty vector control group. Mechanistically, ApoE activates the PI3K/AKT/mTOR signaling cascade to counteract cisplatin cytotoxicity by suppressing cell death and sustaining pro-survival signals.

**Conclusions:**

Overall, this study reveals that M2d TAMs induce platinum resistance in OC cells via exosomes carrying ApoE.

## 1 Introduction

Ovarian cancer (OC) is a lethal gynecological malignancy that is a common cause of morbidity and mortality worldwide, accounting for about 300,000 new cases and more than 150,000 deaths yearly (1, 2). Most patients are treated with both cytoreductive surgery and platinum-based chemotherapy, and the majority of them have a favorable response to platinum-based chemotherapy at an early stage. However, the vast majority of patients will still recur (3, 4), accompanied by platinum resistance. The molecular mechanisms underlying platinum resistance are attributed to multi-factor synergy, including genetic, epigenetic, and microenvironmental factors (5). Herein, the tumor microenvironment (TME) is widely reported as playing a critical role in platinum resistance development and tumor progression (3, 4, 6).

TME consists of stromal cells, blood vessels, extracellular matrix proteins, as well as diverse immune-associated cells including lymphocytes, macrophages, and bone marrow-derived inflammatory cells such as myeloid-derived suppressor cells (MDSCs) and monocytes (7). Tumor-associated macrophages (TAMs) are the major constituent immune cells of the TME in many cancers (8). TAMs in OC can be polarized into two opposing phenotypes during OC progression, including M1 TAMs, exerting antitumorigenic activity, and M2 TAMs, exhibiting protumorigenic activity (9–11). Recent investigations demonstrated that M2 TAMs promote OC cell invasion (12), metastasis (12), and proliferation (13), which is usually correlated with an unfavorable patient prognosis. Furthermore, our previous studies elucidated the mechanism of cisplatin resistance of OC cells via M2 TAMs by enhancing WTAP‐mediated N^6^‐methyladenosine RNA methylation via the CXCL16/CXCR6 axis (14). M2 TAMs, belonging to plastic cells, consist of multiple subsets. Mantovani et al. (15) reported M2a, M2b, and M2c TAMs subsets elicited by IL-4 (or IL-13), IL-1, and IL-10, respectively. In addition, Duluc et al. (16) identified M2d TAMs induced by IL-6. Our preliminary findings reveal that M2d TAMs confer the most potent platinum resistance (**Supplementary Figures S1**, **S2**, in conjunction with a companion study under review). This study therefore set out to explore how M2d TAMs regulate platinum resistance in OC cells.

Recent studies demonstrated that the intercellular communication between TAMs and OC cells confers platinum resistance (13, 17). Exosomes, a subtype of small extracellular vesicles, are well known as mediators in regulating the bidirectional communication between OC cells and TAMs, which consists of miRNAs, proteins, and nucleic acids (17–19). Exosomes secreted by TAMs lead to immune evasion and chemoresistance in OC cells by modulating their adhesion, invasion, and angiogenesis capabilities (20–23). In turn, tumor-derived exosomes can induce TAMs differentiation and phenotypic polarization, a process that further accelerates cancer progression and metastasis (20–24). Apolipoprotein E (ApoE), a highly abundant protein derived from M2d exosomes (**Supplementary Figure S1**), has been widely reported in various neurodegenerative diseases, including Alzheimer’s and Parkinson’s diseases, etc. (25–28). Moreover, accumulating evidence has indicated that ApoE is implicated in DNA synthesis, cell proliferation, angiogenesis, and metastasis (29–33). ApoE overexpression has been correlated with tumor progression and metastasis in multiple malignancies, including gastric, lung, prostate, thyroid, ovarian, and endometrial cancers (29–33). However, no direct evidence has demonstrated the relation of M2d TAMs and ApoE in cisplatin resistance in OC to date.

In this study, M2d TAMs were selected as the cellular source of ApoE to elucidate a potential regulatory role in platinum resistance in OC cells. Using nude mouse xenograft models of human OC and parallel experimental systems, we elucidated the mechanisms by which ApoE and M2d TAMs drive platinum resistance. Moreover, we clarified that they facilitate OC progression through activation of the phosphatidylinositol 3-kinase (PI3K)/AKT/mammalian target of rapamycin (mTOR) pathway.

## 2 Materials and methods

### 2.1 Cell culture and treatment

SKOV3 human OC cells (Procell, Cat. No. CL-0215, Wuhan, China) were cultured in complete growth medium consisting of McCoy’s 5A medium supplemented with 10% fetal bovine serum (FBS) and 1% penicillin-streptomycin (P/S). THP-1 human monocytic cells (Procell) were cultured in RPMI-1640 complete medium supplemented with 10% FBS, 1% P/S, and 0.05 mM β-mercaptoethanol. Both cells were incubated in a humidified incubator at 37 °C with 5% CO_2_. The list of all experimental reagents and materials used in this study was shown in **Supplementary Table S2**.

### 2.2 Macrophage polarization assay

THP-1 cells were induced into M0 TAMs by incubation with medium containing 100 ng/mL phorbol 12-myristate 13-acetate (PMA) for 24 h, and further differentiated into M2d TAMs by incubation with medium containing 30 ng/mL IL-6 for 72 h.

### 2.3 ApoE overexpressing lentivirus construction

ApoE overexpressing lentivirus and control empty vector lentivirus were custom-synthesized by Pricella for infecting SKOV3 OC cells. SKOV3 cells were seeded into 6-well plates. The lentivirus was diluted to a titer of 10^7^ TU/mL. 0.3 mL of the diluted lentivirus solution was added to the cells. 40 μL infection enhancer and 0.3 mL lentivirus were added to the culture medium for viral infection. After incubation at 37 °C for 16 h, the medium was replaced with fresh complete medium for continuous culture. Screening of stable transfected cell lines was conducted at 48 h post-infection. Cells were cultured in medium containing 2 µg/mL puromycin for 48 h. Subsequently, the surviving cells were re-cultured in medium supplemented with 1 µg/mL puromycin for another 48 h.

For lentiviral infection efficiency detection, cells on coverslips were fixed with 4% paraformaldehyde, permeabilized with 0.3% Triton X-100, and counterstained with DAPI. After mounting with anti-fluorescence quenching medium, samples were examined under a laser scanning confocal microscope. The final surviving cells were identified as stable transfected cell lines, as shown in **Figures 1E, F**.

**Figure 1:**
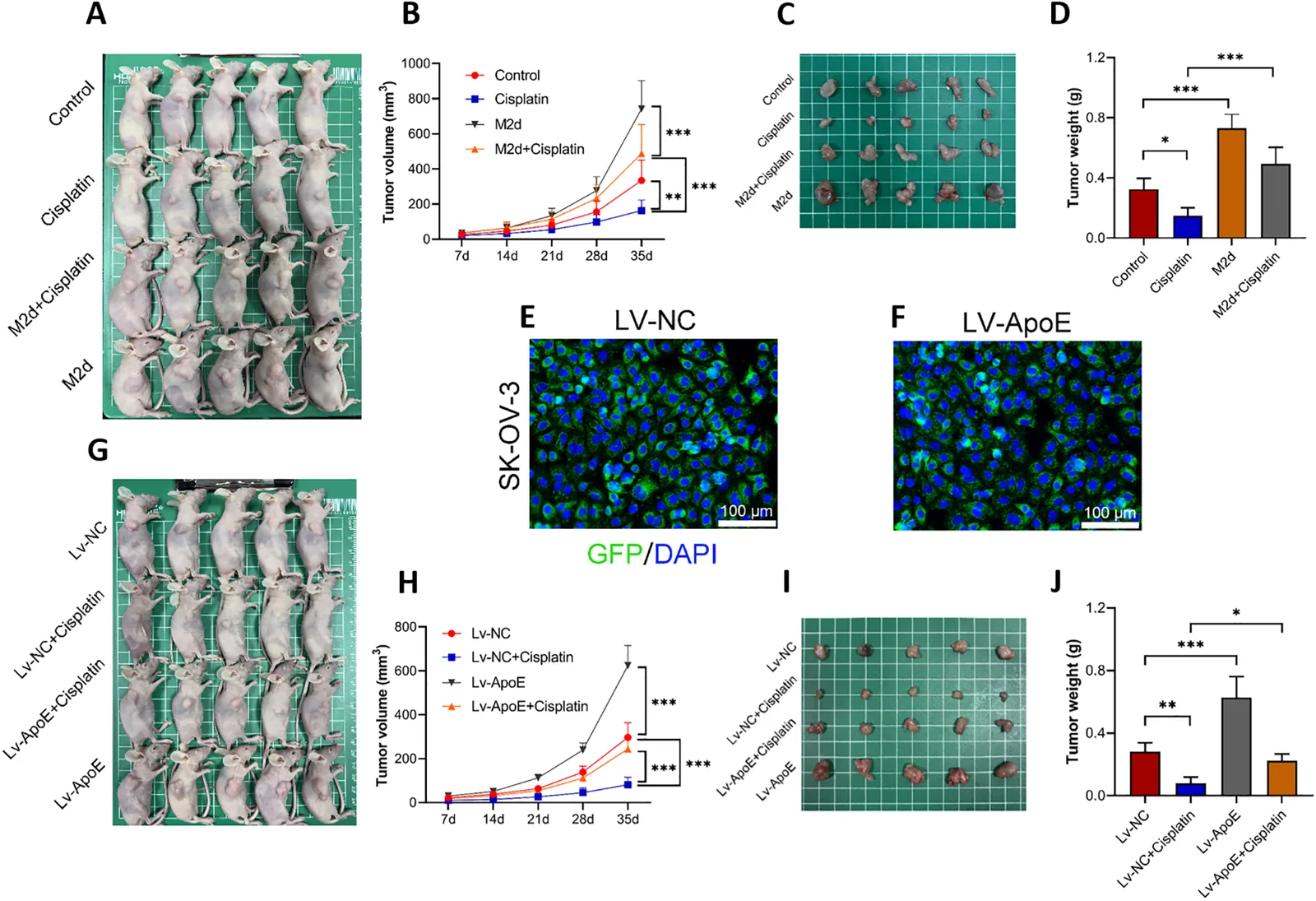
M2d TAMs-derived ApoE drives cisplatin resistance in ovarian xenografts **(A, G)** Subcutaneous transplanted tumors in a whole animal. **(B)** Tumor volume growth curves in: control OC group; cisplatin-treated group; M2d TAMs-OC coinoculation group; and M2d TAMs + cisplatin group. **(E, F)** Lentiviral infection efficiency in SKOV3 OC cells, indicating a high transfection efficiency. Representative laser scanning confocal images showing GFP expression (green) and DAPI-labeled nuclei (blue) in Lv-NC and Lv-ApoE groups. **(H)** Tumor volume growth curves in: Lv-NC group; Lv-NC + cisplatin group; Lv-ApoE treated group; and Lv-ApoE + cisplatin group. **(C, I)** Representative images of excised tumor tissues harvested at the end of the experiment for different groups. **(D, J)** Statistical analysis of excised tumor weights from each group at the experimental endpoint. Data are presented as mean ± SD (n = 5 per group). *P < 0.05, **P < 0.01, ***P < 0.001.

### 2.4 Tumor xenografts in mice

A total of one hundred female specific-pathogen-free (SPF) BALB/c-*Foxn1*^nu^ nude mice (42-56 days old, body weight 17-20 g) were purchased from the Guangdong Medical Laboratory Animal Center (Guangzhou, China; production license No. SCXK (Yue) 2022-0002). This strain carries the *Foxn1*^nu^ loss-of-function mutation.

All animal procedures were performed in strict accordance with the ARRIVE guidelines. Animals were group-housed (4–5 per cage) in a SPF facility under a 12-hour light-dark cycle, with ambient temperature maintained at 22 ± 2 °C and relative humidity kept at 50 ± 10%. During the entire experimental period, animals had ad libitum access to standard laboratory chow and drinking water. Environmental enrichment, including sterilized nesting materials, cardboard tubes and hiding shelters, was supplied to support natural behaviors and enhance animal welfare. Trained staff monitored animals at least twice daily for clinical signs of pain, suffering or distress, such as weight loss, hunched posture, decreased grooming and abnormal vocalization. To alleviate peri-operative discomfort, buprenorphine (0.05 mg/kg) was administered subcutaneously before surgery and every 12 h for 3 days post-operation. Supportive care, including soft palatable food and warming pads for post-surgical recovery, was offered when necessary. Animals that reached pre-defined humane endpoints were euthanized promptly.

SKOV3 cells, as well as a mixture of SKOV3 cells and M2d TAMs at a 1:1 ratio (each cell type at a density of 1×10^7^ cells/mL, 0.2 mL), were inoculated into the axillary fossa of nude mice to establish xenograft tumor models, with five nude mice allocated to each group. Cisplatin was intraperitoneally injected at a dose of 1 mg/kg once a week starting one week after cell inoculation, while the control group was injected with an equal volume of normal saline via the same route. All nude mice were sacrificed by cervical dislocation five weeks later. Death verification was conducted by continuous observation for at least 3 minutes, including confirmation of disappearance of corneal reflex, cessation of spontaneous respiration, and heartbeat to ensure complete death before tumor tissue collection. The tumor tissues were excised at the end of the experiment, with tissue volume calculated by equation *V* = 0.5 × *a* × *b*^2^, where *a* is the longest diameter and *b* is the shortest diameter of the tumor.

### 2.5 RNA extraction and RT-qPCR

To further unveil the relationship between platinum resistance in OC cells and M2d TAMs, total RNA was extracted from appropriate tissue samples. Tissue samples were ground thoroughly in liquid nitrogen, mixed with 1 mL Trizol, vortexed, and incubated at 4 °C for 5 min. 0.2 mL chloroform was added. The mixture was vortexed vigorously for 15 s, incubated at 4 °C for 3 min, and centrifuged at 18400 ×g for 15 min at 4 °C. The upper aqueous phase was transferred to a new centrifuge tube. An equal volume of isopropanol was added and mixed well. The mixture was incubated at -20 °C for 20 min, then centrifuged at 18400 ×g for 15 min at 4 °C. The supernatant was discarded. The RNA pellet was washed with 1 mL 75% DEPC-treated ethanol, centrifuged at 8200 ×g for 5 min at 4 °C. The supernatant was discarded again. The RNA pellet was air-dried at room temperature and dissolved in 30 μL DEPC-treated ddH_2_O. 1.5 μL of the RNA extract was sampled for concentration determination using an ultramicro ultraviolet spectrophotometer. The extracted RNA was stored at -80 °C for subsequent use after concentration quantification. 2 μg of total RNA was used as the template. A 10 μl reverse transcription reaction system was prepared following the manufacturer’s instructions for the Bestar qPCR RT Kit. First-strand cDNA was synthesized. Real-time PCR amplification was performed in a 20 μl reaction system. The system contained DBI Bestar^®^ SybrGreen qPCR MasterMix. Experiments were conducted on an Agilent Stratagene Mx3000P real-time fluorescent quantitative PCR instrument. The sequences of all indicated primers were listed in **Supplementary Table S1**, and representative RT-qPCR amplification curves and melting-curve analysis were shown in **Supplementary Figure S3**.

### 2.6 Hematoxylin-eosin staining

The obtained cancer tissues (on the 35th day, end point of the experiments) were fixed in 4% paraformaldehyde for 24 h. Dehydration was performed through a graded ethanol series. 75% ethanol for 4 h, 85% ethanol for 2 h, 90% ethanol for 2 h, 95% ethanol for 1 h, absolute ethanol twice for 30 min each, followed by absolute ethanol and xylene for 5–10 min each. Tissues were cleared in xylene for 5–10 min and then embedded in paraffin after wax infiltration for 3 h. Sections were cut at a thickness of 4 µm using a microtome and spread on slides. Deparaffinization and rehydration were carried out through xylene twice for 20 min each, 100% ethanol twice for 10 min each, 95% ethanol for 5 min, 90% ethanol for 5 min, 80% ethanol for 5 min, 70% ethanol for 5 min, and finally distilled water for 2 min. Sections were stained with hematoxylin for 3–8 min and rinsed with distilled water to remove excess stain. Differentiation was performed in a differentiating solution for a few seconds, followed by two washes in distilled water. Tissues were blued with 0.6% ammonia water, followed by distilled water rinsing. Counterstaining was done in eosin for 1–3 min. The sections were rapidly dehydrated through a graded ethanol series, cleared in xylene twice for 5 min each, and mounted with neutral balsam. Slides were examined under a microscope.

### 2.7 Apoptosis analysis by terminal deoxynucleotidyl transferase dUTP nick end labeling staining

Paraffin-embedded sections were dewaxed, rehydrated, and treated with Proteinase K for antigen retrieval. TUNEL staining was performed using a commercial kit following the manufacturer’s instructions, with DAPI counterstaining for nuclear visualization. Apoptotic cells were identified by positive TUNEL staining, and images were captured under a fluorescence microscope for quantitative analysis. Apoptotic cells were identified by red fluorescence, with nuclei counterstained blue.

### 2.8 Western blot analysis

Cell lysis was performed at 4 °C using radio-immunoprecipitation assay (RIPA) lysis buffer supplemented with phenylmethanesulfonyl fluoride (PMSF) and phosphatase inhibitors to prevent protein degradation and dephosphorylation. Following lysis, the cell lysates were centrifuged in a 1.5 mL centrifuge tube (equivalent to 25000 ×g with a typical rotor radius) for 10 min at 4 °C, and the resulting supernatants were carefully collected to obtain total cellular proteins. The concentration of total proteins in the supernatants was determined using a bicinchoninic acid (BCA) protein assay kit according to the manufacturer’s instructions. Equal amounts of protein samples (20μl of diluted sample) were separated by sodium dodecyl sulfate-polyacrylamide gel electrophoresis (SDS-PAGE) and subsequently electrotransferred onto polyvinylidene fluoride (PVDF) membranes. PVDF membranes were rinsed with 25 mL TBS for 5 min. Membranes were transferred to blocking buffer (5% nonfat milk in TBS). After blocking, the membranes were incubated overnight at 4 °C with primary antibodies specific to the target proteins at the appropriate dilution.

The target proteins included ApoE, PI3K, total Protein Kinase B (t-AKT), phosphorylated AKT (p-AKT), mTOR, and phosphorylated mechanistic (mammalian) target of rapamycin(p-mTOR). Subsequently, the membranes were rinsed three times with Tris-Buffered Saline with Tween-20 (TBST), with each rinse lasting 5 min to remove unbound primary antibodies. PVDF membranes were placed into a small trough containing 3 mL of horseradish peroxidase-conjugated diluted secondary antibody solution. Membranes were incubated with gentle shaking at room temperature for 40 min. PVDF membranes were washed with 25 mL TBST three times. Each wash lasted for 5 min. Membranes were prepared for subsequent experiments. Finally, the protein bands were visualized using an enhanced chemiluminescence (ECL) detection system, and the images were captured with a gel imaging apparatus.

### 2.9 Statistical analysis

The relative expression level of ApoE was analyzed using the 2^-ΔΔCt^ method. For each sample, ΔCt was calculated as the Ct value of the target gene minus the Ct value of the reference gene. ΔΔCt was calculated as ΔCt of the target gene in experimental samples minus ΔCt of the target gene in calibrator reference samples, and results were presented as mean ± SD. When no predefined calibrator reference sample was available, the sample with the highest Ct value was chosen as the calibrator for calculation. The relative initial template abundance of each sample was expressed as mean ± SD of 2^-ΔΔCt^. For group comparisons, a two-tailed Student’s t-test was used for two-group comparisons, and one-way analysis of variance (ANOVA) was applied for comparisons involving three or more groups. P < 0.05 was considered statistically significant.

## 3 Results

### 3.1 M2d TAMs-derived ApoE mediates cisplatin resistance in ovarian xenografts

To validate the *in vivo* growth of OC and whether M2d TAMs and ApoE overexpression could induce cisplatin resistance in OC cells, a subcutaneous xenograft model of human OC in nude mice was established by axillary injection. **Figures 1E, F** demonstrated high and comparable GFP-positive rates in SK-OV-3 cells transfected with Lv-NC and Lv-ApoE lentiviruses, confirming successful lentiviral transduction with stable and high infection efficiency. These stable cell lines were thus applied for subsequent investigation into the role of ApoE overexpression in cisplatin resistance using a subcutaneous xenograft model. Meanwhile, a mixture of SKOV3 cells and M2d TAMs at a 1:1 ratio was injected as a parallel study to explore their effects on OC tumor growth (**Figure 1A**). **Figures 1B, H** showed that cisplatin monotherapy suppressed tumor growth as expected, while both M2d TAMs and ApoE overexpression accelerated the volume growth of OC tumors compared to the OC control group. Comparison of tumor growth curves indicated that M2d TAMs and ApoE overexpression both blunted the anti-tumor activity of cisplatin. Elevated proliferation was observed in cisplatin-exposed OC cells upon M2d TAMs treatment or ApoE overexpression, which further verified the development of cisplatin resistance. All nude mice were humanely euthanized at the experimental endpoint, and tumor tissues were excised meticulously for subsequent analysis as shown in **Figures 1C, D**. Weight analysis showed that M2d TAMs and ApoE overexpression exert a prominent promotional effect on tumor progression (**Figures 1G, H**), which is consistent with the volume comparison result. Taken together, xenograft model results demonstrated that M2d TAMs and ApoE overexpression facilitate the proliferation of OC xenografts *in vivo*, and might further confer chemoresistance to cisplatin in OC cells.

### 3.2 ApoE overexpression mitigates cisplatin-induced apoptosis in ovarian tumor tissues

To elucidate the regulatory role of M2d TAMs and ApoE overexpression on cell proliferation and apoptosis in OC xenografts, HE staining and TUNEL staining were performed. HE staining (**Figures 2A, D**) showed tissue damage in both cisplatin treated groups, accompanied by extensive cell necrosis, nuclear pyknosis, inflammatory cell infiltration, and disordered cell arrangement as expected. For M2d TAMs or Lv-ApoE groups, the cells were regularly arranged, the degree of necrosis was mild, no obvious tissue damage was observed, and the cell number was markedly elevated compared to control groups, respectively. For groups treated with cisplatin, either M2d + Cisplatin or Lv-ApoE + Cisplatin group could attenuate cisplatin-induced tissue injury in OC, accompanied by an increased number of surviving OC cells. Collectively, the HE staining results indicated that both M2d TAMs and ApoE overexpression could promote the proliferation of OC cells and inhibit cell apoptosis to a certain extent.

**Figure 2:**
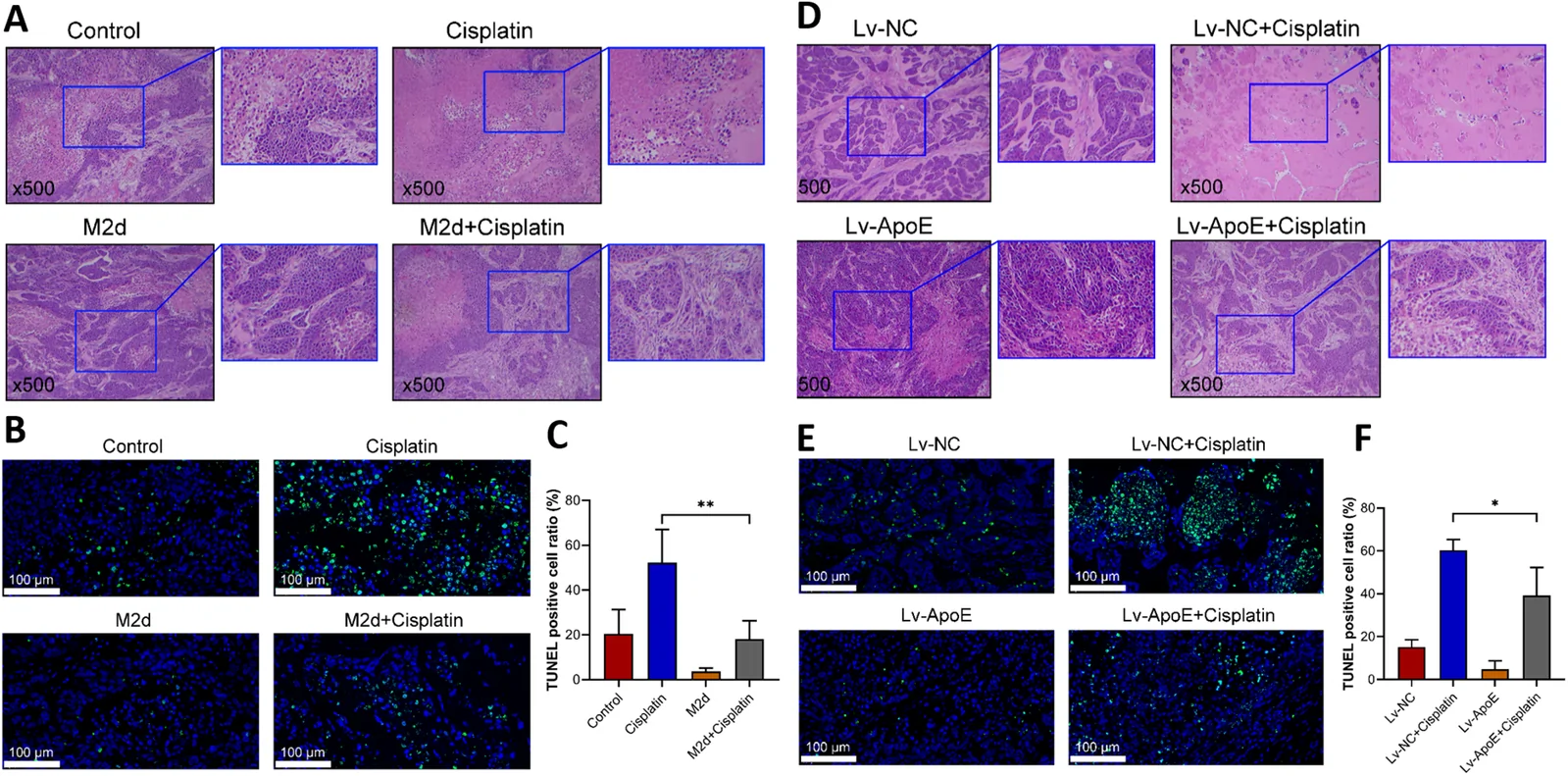
Histological assessment of tumor apoptosis in OC xenografts modulated by M2d TAMs and ApoE overexpression. **(A, D)** Representative HE-stained xenograft tumor sections. **(B, E)** TUNEL staining for apoptotic cells (green) and DAPI-labeled nuclei (blue) in tumor tissues. **(C, F)** Statistical quantification of TUNEL-positive cell percentages. Data are mean ± SD. *P < 0.05, **P < 0.01.

TUNEL staining results (**Figures 2B, C, E, F**) exhibited that cisplatin treatment promotes OC cell apoptosis, while M2d TAMs and ApoE overexpression suppress OC cell apoptosis, as evidenced by a lower apoptotic rate relative to the control group, respectively. Moreover, either M2d TAMs or ApoE overexpression can reduce the number of apoptotic cells in OC tissues upon cisplatin exposure. Taken together, these results indicated that M2d TAMs and ApoE overexpression inhibited cisplatin-induced apoptosis of OC cells and promoted proliferation *in vivo*, thereby attenuating cisplatin-mediated tissue damage. Collectively, ApoE overexpression and M2d TAMs exhibited equivalent biological functions in the above experiments.

### 3.3 M2d TAM-derived ApoE triggers PI3K/AKT/mTOR signaling to sustain OC cisplatin resistance

ApoE expression has been proven to be indispensable for the proliferation and survival of OC cells (34). In addition, pan-cancer profiling of ApoE demonstrates that its genetic aberrations participate in the concerted modulation of estrogen signaling and the PI3K/Akt/mTOR cascade, alongside other biological processes. They also correlate closely with clinical prognosis and tumor immune infiltration in patients (35). The Cancer Genome Atlas (TCGA) has revealed extensive molecular heterogeneity in OC, along with frequent dysregulation of key signaling pathways. The PI3K/AKT/mTOR pathway, a well-characterized oncogenic signaling cascade, harbors mutations or amplifications in a high proportion of OC cases (36), and its aberrant activation is critical for driving OC progression (37). Herein, we investigate the correlation between M2d/ApoE and the PI3K/Akt/mTOR signaling pathway in OC.

Furthermore, it should be noted that this TME-M2d TAM-ApoE-PI3K/AKT/mTOR regulatory axis is not exclusive to cisplatin. For example, among clinically common chemotherapeutic agents for OC, carboplatin is another typical alternative drug that can completely activate the entire resistance pathway. As another mainstream platinum chemotherapeutic sharing an identical DNA cross-linking cytotoxic mode with cisplatin, carboplatin also triggers immunosuppressive remodeling of the TME, drives M2d polarization of TAMs, elevates ApoE levels, and activates downstream PI3K/AKT/mTOR signaling to mediate cross-resistance (38). In broader terms, any pharmaceutical compound that interferes with any single molecular or cellular link in this cascade (including agents that alter TME immune status, modulate TAM polarization, regulate ApoE expression, or intervene in PI3K/AKT/mTOR cascade activity) is theoretically capable of altering the degree of chemoresistance induced by this pathway. Follow-up independent research will be required to screen and validate candidate targeted or chemotherapeutic drugs capable of reversing or bypassing this M2d TAM-ApoE-dependent resistance mechanism.

**Figures 3A, B** demonstrated that ApoE mRNA levels remained low in both control and Lv-NC groups, with no obvious upregulation following cisplatin treatment. By contrast, M2d TAM intervention and ApoE overexpression significantly elevated ApoE mRNA expression. These results suggested that M2d TAMs constitute the predominant source of ApoE, with cisplatin exerting no regulatory effect on ApoE secretion. OC tissues exhibited phosphorylation of PI3K/AKT/mTOR signaling-associated proteins in the presence of M2d TAMs and ApoE overexpression (**Figures 3C–E**), whereas no significant differences in the total protein levels of PI3K, AKT, and mTOR were observed among experimental groups. Phosphorylated counterparts of these pathway proteins were minimally detected in control and cisplatin treated groups. Collectively, the consistent oncogenic effects of M2d TAMs and ApoE overexpression in these parallel experiments validated that M2d TAMs confer cisplatin chemoresistance via ApoE secretion. Secreted ApoE further triggers activation of the PI3K/AKT/mTOR signaling cascade, thereby facilitating OC cell proliferation, tumorigenesis, and metastatic progression.

**Figure 3:**
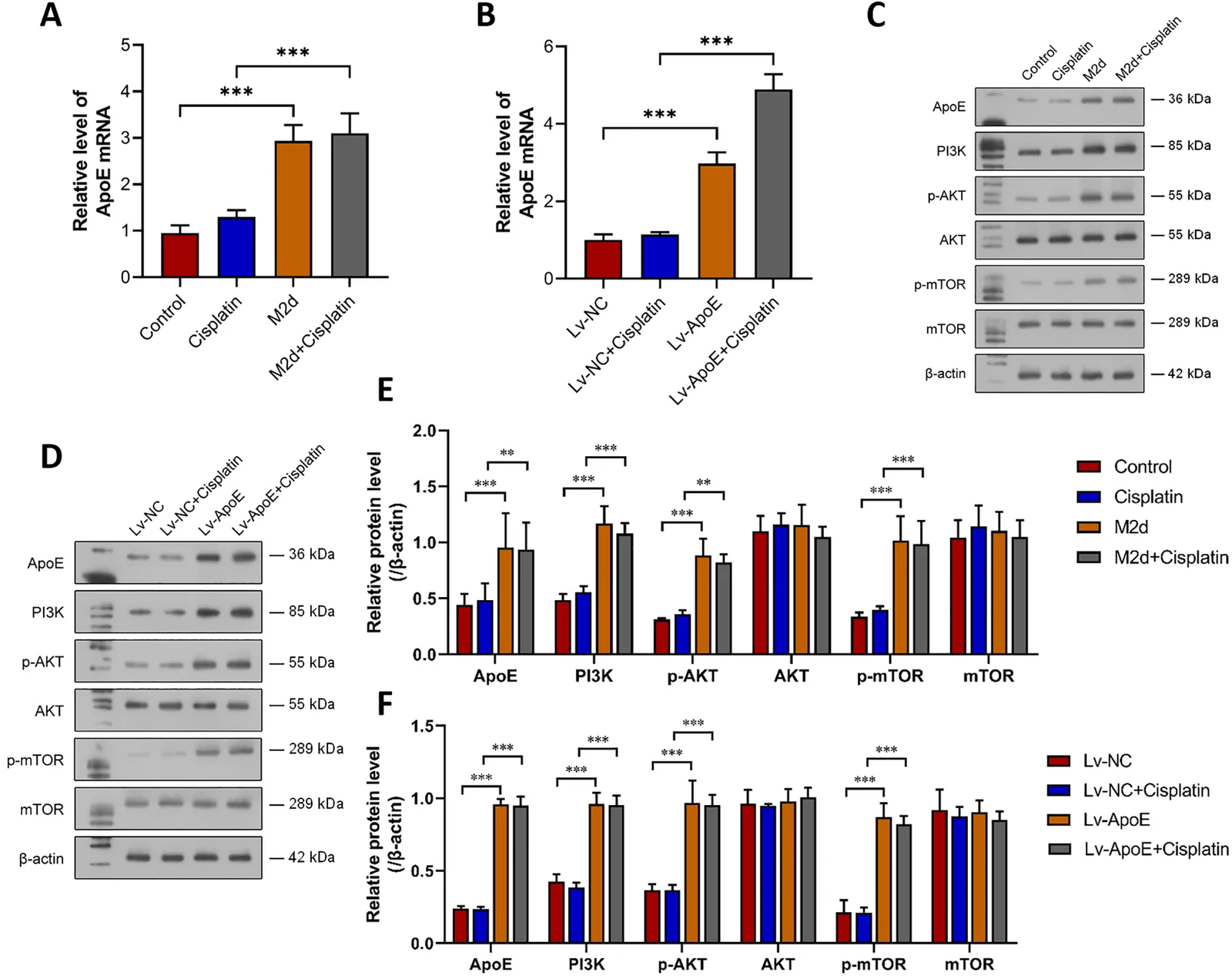
M2d-TAM ApoE activates PI3K/AKT/mTOR to induce OC cisplatin resistance. **(A, B)** Relative mRNA levels of ApoE in different groups were quantified by qRT-PCR. **(C, D)** Representative western blot images of ApoE and PI3K/AKT/mTOR pathway-related proteins in the M2d-TAM-treated and ApoE-overexpressing groups, respectively, with β-actin as the internal loading control. **(E, F)** Quantitative analysis of relative protein expression levels. Data are presented as mean ± SD. **P < 0.01, ***P < 0.001.

## 4 Discussion

Platinum-based chemotherapy remains the first-line and most widely administered regimen for OC, achieving an initial response in approximately 70% of patients (39, 40). While most patients ultimately develop platinum resistance, leading to recurrence and dismal prognosis (41). Despite intensive research over the past decades, the molecular mechanisms underlying platinum resistance in OC remain unclear. Broadly, platinum resistance can be categorized into intracellular mechanisms of platinum resistance and alterations in the tumor microenvironment. For intracellular mechanisms, recent reports indicated that cisplatin exerts its chemotherapeutic effects by binding to nuclear DNA to form platinum-DNA adducts (42). These adducts activate the cellular DNA damage response (DDR): the core sensor kinases ataxia-telangiectasia-mutated (ATM), ataxia-telangiectasia-mutated and Rad3-related (ATR), and DNA-dependent protein kinase catalytic subunit (DNA-PKs) become activated and phosphorylate downstream repair proteins to stabilize stalled replication forks and promote DNA repair, ultimately triggering cell-cycle arrest and apoptosis (43). Dysregulation of the ATM/ATR/DNA-PKs signaling pathway suppresses cisplatin-induced apoptosis (44, 45). Overall, intracellular mechanisms of platinum resistance involve impaired formation of platinum-DNA adducts, as well as functional defects in DNA repair, apoptosis, and cellular metabolism.

In addition, emerging evidence now highlights the TME, particularly TAMs, as pivotal extrinsic regulators of platinum resistance (46, 47). TAMs remodel the TME via cytokines, chemokines, and extracellular vesicles to impair chemotherapy efficacy. Consistent with the well-established paradigm of macrophage polarization, M1 TAMs exert robust antitumor effects in OC by secreting pro-inflammatory cytokines, triggering immunogenic cell death, and suppressing tumor proliferation and metastasis (48–50). Mechanistically, M1 TAMs have been shown to sensitize OC cells to platinum-based chemotherapy by promoting apoptotic signaling (51, 52). In contrast, M2 TAMs are widely recognized as key promoters of OC progression, immune suppression, and therapeutic resistance (53, 54). Our previous study demonstrated that M2d TAMs play a major role in platinum resistance in OC. (**Supplementary Figures S1**, **S2**). However, the specific role of M2d TAMs in driving platinum resistance has not been validated *in vivo*. This investigation demonstrated that the existence of M2d TAMs accelerated tumor growth and reduced cisplatin chemosensitivity, as objectively validated by increased tumor volume and weight in subcutaneous xenograft models receiving combined cisplatin treatment. Notably, our study demonstrated in a living animal model that the presence of M2d TAMs directly promotes the development of platinum resistance in OC. These data demonstrate that M2d TAMs are one of the key *in vivo* contributors to platinum resistance in OC. Importantly, we also acknowledge that the potential contributions of other uninvestigated cell types cannot be excluded (55). Exosomes act as critical mediators of intercellular communication within the TME (56), and ApoE has been increasingly recognized as a key signaling molecule implicated in malignant progression and therapeutic resistance across multiple cancer types (32, 57–59). To further delineate the molecular mechanism by which M2d TAMs facilitate platinum resistance in OC, we conducted parallel *in vivo* experiments using subcutaneous xenograft models. Our results demonstrated that ApoE exerted consistent pro-tumor and pro-chemoresistance effects comparable to those of M2d TAMs in living animals. Notably, pathway-specific evidence revealed that both ApoE overexpression and M2d TAMs markedly elevated the phosphorylation levels of AKT and mTOR. These findings provided critical *in vivo* evidence supporting that M2d TAMs drive platinum resistance by delivering ApoE as a pivotal signaling messenger to OC cells.

The PI3K/AKT/mTOR pathway represents a canonical signaling cascade that governs anti-apoptotic responses and cell proliferation (37). We demonstrated that activation of this pathway directly antagonizes the cytotoxic effects of cisplatin by suppressing apoptosis and enhancing survival signaling, thereby clarifying the molecular basis underlying ApoE-mediated chemoresistance in OC (60). Nevertheless, the present study does not exclude the potential synergistic involvement of other signaling pathways in this process. This finding bears important clinical implications for the treatment of platinum-resistant OC. Targeting the M2d TAM–exosome–ApoE axis may represent a promising strategy to reverse chemoresistance. Potential interventions include blocking exosome biogenesis or secretion using GW4869 or neutral sphingomyelinase inhibitors (61), and neutralizing exosomal ApoE with anti-ApoE antibodies or competitive peptides (62). Moreover, exosomal ApoE levels in ascites or plasma, together with M2d-specific gene expression in tumor tissues, could serve as novel biomarkers for predicting platinum chemosensitivity and evaluating prognosis in OC patients.

In summary, using two complementary animal models, our study provided *in vivo* evidence that M2d TAMs drive platinum chemoresistance in OC by secreting ApoE-containing exosomes. This exosomal ApoE activates the PI3K/AKT/mTOR signaling pathway in OC cells, thereby inhibiting apoptosis and promoting proliferation. This work establishes a novel mechanism underlying macrophage-mediated chemoresistance and offers a potential target for overcoming treatment failure in OC.

## Data Availability

The original contributions presented in the study are included in the article/**Supplementary Material**. Further inquiries can be directed to the corresponding authors.
