# Supplementary material for: Regulatory mechanism of M2d tumor-associated macrophage-derived exosomal apolipoprotein E on platinum resistance in ovarian cancer
Source: Front Oncol. 2026 Oct 15;16:1946625. doi: 10.3389/fonc.2026.1946625 (PMC13716620; doi:10.3389/fonc.2026.1946625)
Supplement: Supplementary file 1 [file DataSheet1.pdf]

Supplementary Information for: Chunmei Wang et al.

1. Subtype of M2 TAMs ApoE expression

To further unveil the relationship between platinum resistance in OC cells and specific subsets of M2 TAMs, we isolated four M2 TAMs (M2a-d) by flow cytometry sorting, and then the different induced M2 TAM subsets were co-cultured with OC cells in Transwell inserts respectively. Exosomes derived from all M2 subsets TAMs were isolated separately, and the contained proteins were analyzed by a cytokine protein array approach. Our previous investigations revealed that M2d TAMs are the primary source of ApoE secretion, compared with the other three subsets (Figure S1).

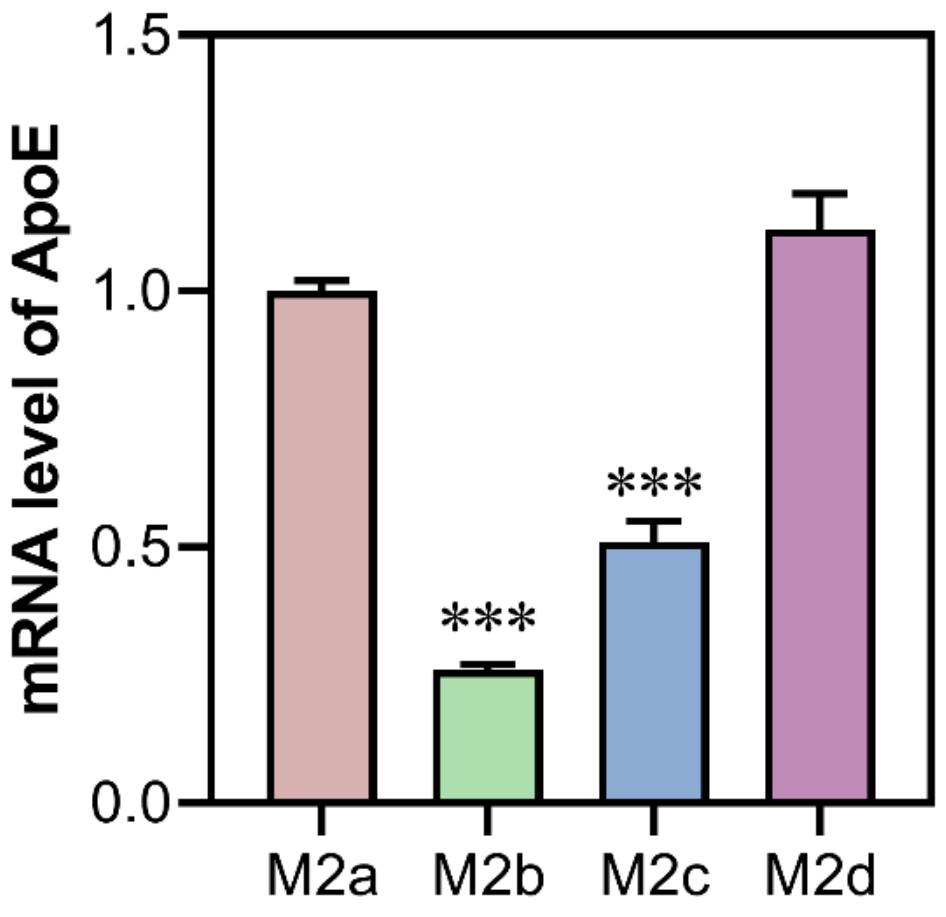

Figure S1. Relative ApoE mRNA expression levels in M2a, M2b, M2c and M2d macrophage subsets. qRT-PCR analysis was performed to detect the relative mRNA level of ApoE in four subtypes of M2 macrophages. Data are presented as mean ± SD. ***$P < 0.001$.

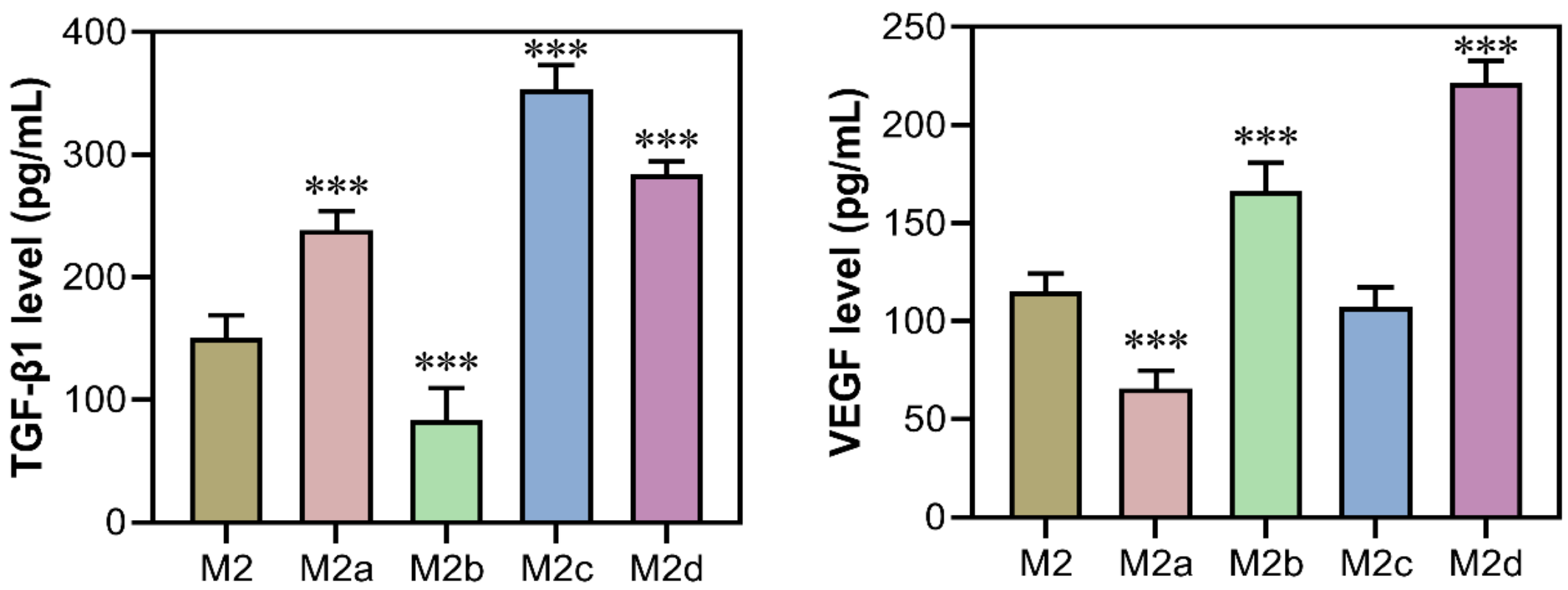

Figure S2. (left) Concentrations of TGF-β1 in the supernatant of different subtype cells before and after stimulation (***P < 0.001). (right) Concentrations of VEGF in the supernatant of different subtype cells before and after stimulation (***P < 0.001).

Moreover, the expression levels of TGF-β1 and VEGF were determined by ELISA, as presented in Figure S2. M2d TAMs displayed high secretion of both cytokines compared with other subtypes, suggesting that M2d TAMs play more substantial roles in promoting vascular endothelial growth, immunosuppression, fibrosis, and tumor microenvironment remodeling. Taken together, these findings support our selection of M2d as the major study subject, based on which we established a subcutaneous xenograft model. This provides a mechanistic rationale for the choice of experimental subjects in subsequent cisplatin-resistance investigations.

2. RT-qPCR for mRNA expression detection

Table S1. Primer sequences for qPCR

Primer	Sequence(5’- 3’)
H-GAPDH-F	TGTTGCCATCAATGACCCCTT
H-GAPDH-R	CTCCACGACGTACTCAGCG
ApoE-F	CGGACATGGAGGACGTGTG
ApoE-R	TTCTGCAGGTCATCGGCATC

ApoE was selected as the target gene for mRNA quantification, and human-derived GAPDH was applied as the reference gene for mRNA normalization. The full thermal cycling protocol was set as follows: initial denaturation at 95 °C for 2 min, followed by 40 cycles of amplification. Each amplification cycle consisted of denaturation at 94 °C for 20 s, annealing at 58 °C for 20 s, and extension at 72 °C for 20 s. Following amplification, melting curve analysis was performed to verify the specificity of PCR products. The melting curve procedure was programmed as 94 °C for 30 s, 65 °C for 30 s, and 94 °C for 30 s. Representative amplification and melting curves are presented in the supplementary materials.

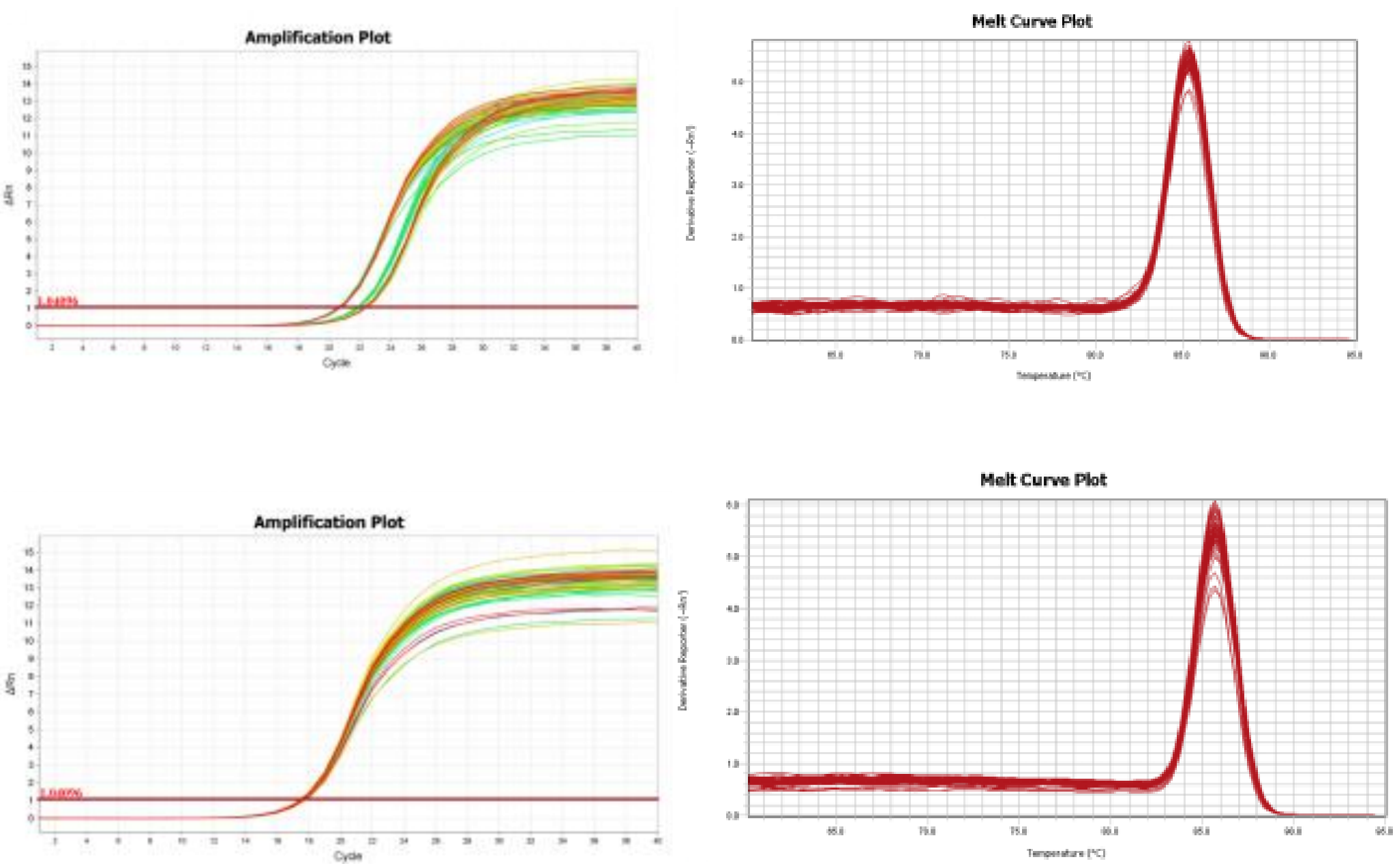

Figure S3. Representative RT-qPCR amplification curves and melting-curve analysis. Left: Amplification plot illustrating △Rn fluorescence as a function of cycle number for ApoE (up) and GAPDH (down). Right: Melting-curve analysis was performed to verify amplification specificity for ApoE (up) and GAPDH (down).

The single distinct melting peak confirms the generation of a homogeneous target amplification and the absence of detectable primer-dimer formation or off-target amplification. Quantitative RT-qPCR in this study adopts the relative quantification strategy, in which the expression of each target gene is normalized to its corresponding reference gene within the same sample to obtain relative expression values for inter-group comparison.

Table S2. List of experimental reagents and materials used in this study. The catalog numbers and antibody working dilutions are presented in the Notes column. — indicates that catalog numbers were not provided by the manufacturer.

**Material**	**Manufacturer**	**Location (City, Country)**	**Notes**
Trizol (9109)	TAKARA	Dalian, China	—
Bestar™ qPCR RT kit (2220)	DBI	Shanghai, China	—

Bestar™ qPCR MasterMix (2043)	DBI	Shanghai, China	—
DEPC (6079)	MACKLIN	Shanghai, China	—
Tris-base, Glycine, SDS	Shanghai Sangon Biological Engineering Co., Ltd.	Shanghai, China	—
Acrylamide, BIS-Acrylamide	Genebase	Shanghai, China	—
BCA	Beyotime	Shanghai, China	—
Ammonium persulphate	GENVIEW	Jacksonville, FL, USA	—
phenylmethylsulfonyl fluoride, Bromophenol blue	Genebase	Shanghai, China	—
TWEEN-20	MP Biomedicals	Irvine, CA, USA	—
DTT	Weijia Technology	Shanghai, China	—
NaCl	Life science&products services	Shanghai, China	—
Lysate	Beyotime	Shanghai, China	—
BSA	Amresco	Solon, OH, USA	—
TEMED	Jingxin Biotechnology Co., Ltd.	Shanghai, China	—
Protein Quantification Kit	Thermo Fisher Scientific	Waltham, MA, USA	—
Methanol, Glycerol, Ethanol, Isopropanol, Concentrated HCl	Guangzhou Chemical Reagent Factory	Guangzhou, China	—
Luminescent liquid	Millipore	Burlington, MA, USA	—
Developer powder, Fixer powder	Tianjin Nankai District Chengxin Medical Auxiliary Materials Factory	Tianjin, China	—
anti-APOE	Servicebio	Wuhan, China	GB115743, 1:1000
anti-PI3K	Servicebio	Wuhan, China	GB11525, 1:1000

anti-AKT	Servicebio	Wuhan, China	GB15689, 1:1000
anti-p-AKT	Servicebio	Wuhan, China	GB150002, 1:1000
anti-mTOR	Servicebio	Wuhan, China	GB11405, 1:1000
anti-p-mTOR	Servicebio	Wuhan, China	GB114686, 1:1000
anti-GAPDH	Servicebio	Wuhan, China	GB11002, 1:1000
HRP Goat anti-Rabbit IgG	Servicebio	Wuhan, China	GB23303, 1:10000
Western Protein Marker	Servicebio	Wuhan, China	G2086
fetal calf serum	Gibco	Grand Island, NY, USA	Cat.No. 10099-141
McCoy's 5A Medium	Pricella	Wuhan, China	Cat.No. PM150710
RPMI-1640 Complete Medium	Pricella	Wuhan, China	Cat.No. PM150110
pancreatin	GENVIEW	Jacksonville, FL, USA	Cat.No. 9002-07-7
mercaptoethanol	MedChemExpress	Monmouth Junction, NJ, USA	Cat.No. HY-Y0326
phorbol-12-myristate-13-acetate(pma)	Servicebio	Wuhan, China	Cat.No. G2272
IL-6	Beyotime	Shanghai, China	Cat.No. P5138
cisplatin	Beyotime	Shanghai, China	Cat.No. Y001313
